# Characteristics of adult individuals with Rett syndrome treated or untreated with trofinetide in the United States

**DOI:** 10.3389/fneur.2026.1856312

**Published:** 2026-07-27

**Authors:** Nazia Rashid, Alyssa Peckham, Vinod Kumar Yakkala, Louise Cosand, Krithika Rajagopalan

**Affiliations:** 1 Medical Affairs, Acadia Pharmaceuticals Inc, San Diego, CA, United States; 2 Anlitiks Inc, Windermere, FL, United States

**Keywords:** adults, Rett syndrome, treated, trofinetide, untreated, real-world evidence

## Abstract

**Background:**

Rett syndrome (RTT) is a rare, progressive MECP2-related neurodevelopmental disorder with substantial lifelong morbidity that persists into adulthood. Although survival has improved, adults often experience evolving multisystem complications and fragmented transition care. Trofinetide (TROF) is approved for ages ≥2 years old, yet data on adults with RTT in the real-world setting remains limited; this study evaluated demographics and characteristics of adults >20 years of age who are treated vs. untreated with TROF.

**Methods:**

A retrospective analysis of individuals with RTT diagnosis (ICD-10-CM: F84.2) from a linked medical claims and specialty pharmacy database from 01/01/2021 to 09/30/2024 was conducted. RTT individuals were categorized into two groups based on treatment status: treated group (index date: 1st TROF prescription (RX) 04/01/2023 to 03/31/2024) and untreated group (index date: assigned date using a risk set sampling method). Individuals who were ≤20 years of age at index date or had diagnosis for brain trauma or cerebrovascular disease prior to RTT diagnosis were excluded. RTT individuals were required to have continuous enrollment for ≥6 months pre-index and post-index. Demographics and clinical characteristics were assessed during pre-index among the treated and untreated groups. Continuous variables were summarized as means and SD; categorical variables as counts and percentages.

**Results:**

There were 1,820 adult RTT individuals (>20 year old) eligible for the analysis: treated group (*n* = 290 [15.9%]) and untreated group (*n* = 1,530 [84.1%]). Mean age (SD) at index date was 30.9 (9.9) years (treated group) vs. 33.5 (10.0) years (untreated); and 5.5% vs. 6.7% were males in the treated vs. untreated groups, respectively. Treated group had higher rates of differential diagnoses, but similar rates of baseline comorbidities among both. Treated group also had higher rates of RTT related clinical features vs. untreated group.

**Conclusion:**

In this real-world analysis, only 16% of eligible adult RTT individuals were initiated on TROF, while 84% remain untreated. There is a high unmet need for adult individuals with RTT to initiate treatment with TROF. Treated group had higher rates of RTT related clinical features and differential diagnoses; however, the observation that TROF is being used in adults with greater to similar baseline complexity compared to untreated may provide reassurance for prescribers to consider TROF in adults who were untreated.

## 1 Introduction

Rett syndrome (RTT) is a rare, progressive neurodevelopmental disorder that primarily affects females and is most commonly associated with pathogenic variants in the methyl-CpG-binding protein 2 (MECP2) gene. Typical RTT is generally associated with MECP2 mutations; however, atypical RTT or RTT-like neurodevelopmental phenotypes may be associated with other genes, including CDKL5 and FOXG1 (1, 2). Deficiency in the MECP2 protein disrupts normal neuronal development and synaptic structure and neuron function, leading to loss of acquired purposeful hand skills and spoken language along with development of gait abnormalities, and hand stereotypies (3–5). Compounding this, the patients also develop clinical complications and comorbidities such as seizures, and severe gastrointestinal dysfunction among others (4, 5). Overall, RTT and associated comorbidities contribute to substantial lifelong morbidity and higher health care resource utilization burden (6, 7).

Historically, RTT was viewed as a pediatric condition; with symptoms emerging in early childhood (usually 6–18 months) that persist throughout a person’s life (8). Although it is a debilitating and chronic condition, advances in multidisciplinary supportive care have significantly increased life expectancy among individuals with RTT; with nearly 60% individuals now surviving well into their fourth and fifth decades (9–11). Although little has been known about the aging process of RTT, recent research has attempted to examine potential differences between younger individuals with RTT vs. adults with RTT. For example, a 15-year longitudinal natural history study of caregiver-provided historical and clinically observed RTT symptoms reported that older individuals (aged ≥30 years) reported similar severity levels of RTT as younger individuals (aged <30 years). However, the older individuals reported worsening in loss of motor features and functioning skills, but improvements in nonverbal communication compared to younger individuals (12). These findings are consistent with other research which suggests that adults with RTT may face significant motor skill loss, worsening scoliosis, breathing difficulties, and severe epilepsy (13–15). Overall, younger RTT individuals who transition into adulthood often face significant challenges in continuity of care and decline in specialized multidisciplinary support, even as the burden of chronic comorbidities such as epilepsy and scoliosis may evolve (16, 17).

In the United States (US), real-world evidence using administrative claims data indicates that a substantial proportion of individuals may have their first observed RTT diagnosis recorded in adulthood (≥18 yrs) (7). In this study, it is possible that a majority of adults may have been born before the turn of 21st century and potentially faced challenges in getting an appropriate diagnosis in childhood due to potential challenges in differentiating RTT from similar disease states. The discovery of the MECP2 gene in 1999 and the development of defined RTT diagnostic criteria in 2010 have changed the screening and diagnostic landscape of RTT to include both children and adults in diagnostic testing. Therefore, it is important for clinicians and caregivers to recognize the clinical features of RTT among adults to avoid delays in diagnosis and ensure timely initiation of treatment. Currently, trofinetide (TROF) is the first and only treatment for RTT for all individuals aged ≥2 years (18–21). The primary clinical trial supporting the efficacy and safety of TROF in RTT was the 12-week, phase 3, placebo-controlled LAVENDER study in females with RTT aged 5–20 years (22); participants who completed the study could continue treatment in LILAC and LILAC-2, 40-week and 32-month open-label extension studies of LAVENDER, respectively (23, 24). Additionally, the tolerability and efficacy of TROF in girls with RTT aged 2–4 years was assessed in the open-label phase 2/3 DAFFODIL study (25). The only clinical trial that evaluated the efficacy and safety of TROF among adult patients (>20 years of age) with RTT aged into their forties was an early phase 2 trial (26); however, the dosing in this trial was lower than that of subsequent trials. In summary, LAVENDER, LILAC, LILAC-2, and DAFFODIL included females aged 2–20 years only; thus, there is a critical need to better understand the demographic and clinical characteristics of adults >20 years of age who initiate TROF for the treatment of RTT in real-world settings. To address this need, a real-world study was conducted to examine patient demographics and clinical characteristics among adult RTT individuals who were either treated or untreated with TROF in clinical practice.

## 2 Methods

### 2.1 Study design, data source, and population

A retrospective cohort study was conducted using linked data from two claims data sources: (i) IQVIA’s Anonymized Patient Level Data (APLD), and (ii) specialty pharmacy data consisting of TROF prescription (RX) and dosing information. The APLD is an open-source, administrative claims data that includes pre-adjudicated, de-identified healthcare claims data for over 130 million beneficiaries in the US. The database contains detailed information on demographics, medical claims such as diagnosis, procedures, services, and pharmacy claims, making it a robust real-world data resource to complete the study objectives. The specialty pharmacy data contains dispensing information about TROF treatment among individuals with RTT including date of dispensing, quantity dispensed, days of supply etc.

The eligible study population included individuals with at least one medical claim for RTT (ICD-10-CM: F84.2) in any diagnostic position from 01/01/2021 to 09/30/2024 (i.e., study period). Individuals were categorized with a TROF RX (treated group) vs. no TROF RX (untreated group). To be included, all individuals in both the treated and untreated groups were required to be >20 years of age at their index date and to have continuous enrollment for ≥6 months pre-index and post-index. Individuals who did not meet the continuous-enrollment requirement were excluded from the analytic sample. Individuals with a diagnosis of cerebrovascular disease (I60.x–I69.x) or brain trauma (S06.x) on or before RTT diagnosis were also excluded.

The index date was defined as the first TROF RX claim for the treated group during the identification period (04/01/2023 to 03/31/2024). An index date for the untreated group was needed to ensure that the TROF unexposed population (i.e., untreated group) and the TROF-treated adults were comparable. To create an index date for the untreated group, a risk set sampling approach was conducted which also helped reduce immortal time bias (27, 28). For each individual in the treated group, the elapsed time (in days) from RTT diagnosis to TROF initiation was calculated. A “risk set” of untreated individuals who (i) had a RTT diagnosis, and (ii) had not initiated TROF was then identified. From this eligible risk set, five untreated individuals were sampled and assigned a proxy-index date equal to their RTT diagnosis date plus the treated individual’s diagnosis-to-treatment time (1:5 ratio) (27, 28). This process was repeated for all treated individuals to create comparable person-time between treated and untreated groups. The untreated proxy-index date was defined as: untreated RTT diagnosis date + treated diagnosis-to-TROF time (days).

### 2.2 Study measures

Baseline demographics and clinical characteristics were assessed during the 6-month pre-index period among both treated and untreated cohorts. Demographic variables evaluated at index date included age, sex (male/female), and geographic region (Midwest, Northeast, South, West). In addition to age at index, age at RTT diagnosis was also assessed. Clinical characteristics included differential diagnosis (e.g., autism spectrum disorder (ASD), non-specific developmental delay (NSDD) etc.), comorbidities (e.g., epilepsy, seizures, scoliosis, gastrostomy) and RTT- related clinical features (e.g., loss of acquired communicational skills, hand apraxia/dyspraxia). Physician specialty (e.g., child neurologist, neurologist, pediatrician) was determined based on the claim closest to the index date.

### 2.3 Statistical analyses

Demographics and clinical characteristics were reported as frequencies and percentages (%) for categorical variables; mean and standard deviation (SD) for continuous variables. Between-group comparisons were conducted using two sample t-tests for continuous variables and chi-square tests for categorical variables; Fisher’s exact tests were used when expected cell counts were small (*n* < 5). A *p*-value of <0.05 was considered statistically significant and all analysis was conducted using Anlitiks’ proprietary RapidAnalyzer™ analytic platform that is powered by SQL and RStudio.

## 3 Results

### 3.1 Study population

**Figure 1:**
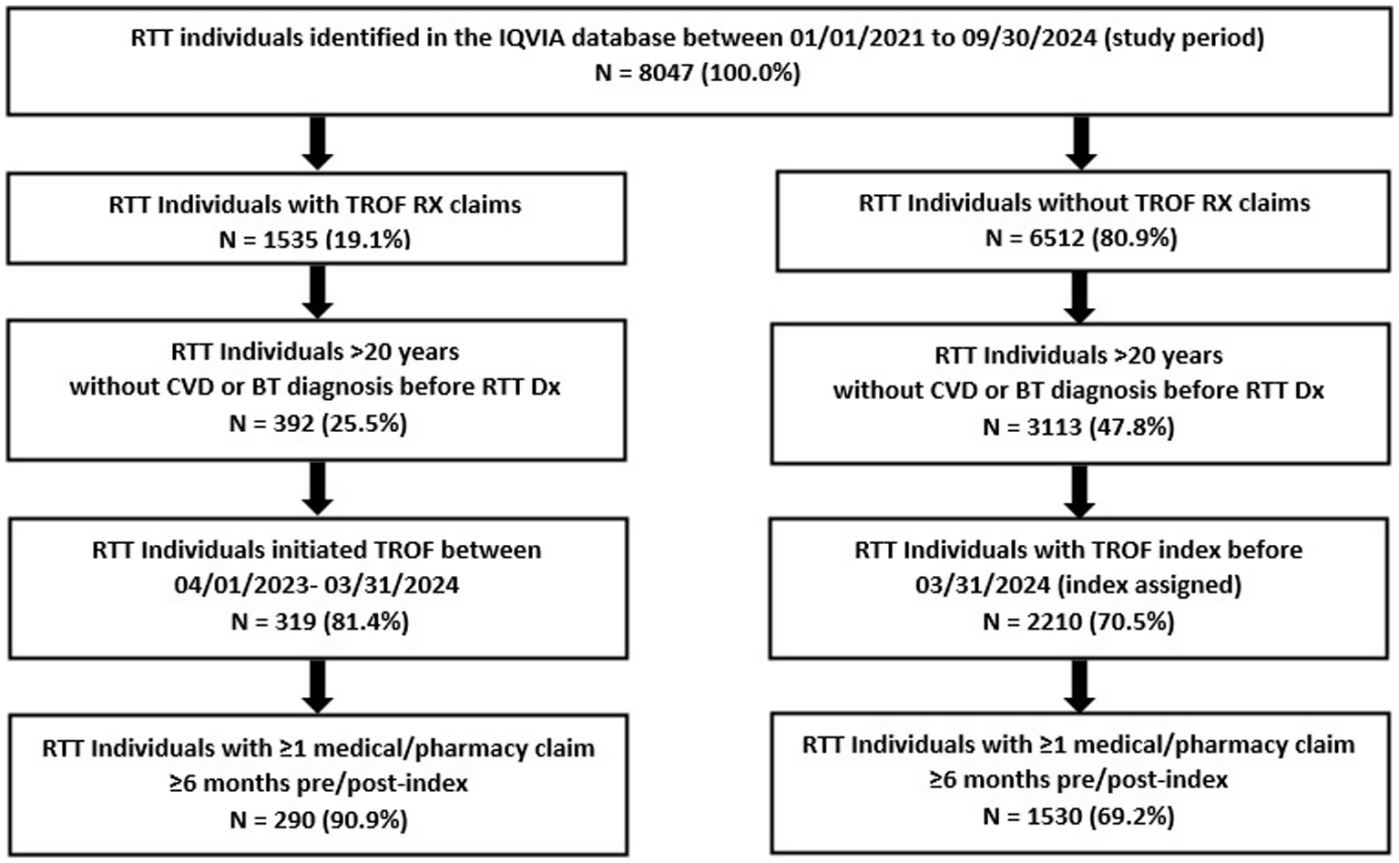
Study attrition. BT, brain trauma; CVD, cerebrovascular disease; RTT, Rett syndrome; TROF, trofinetide; Dx, diagnosis. The percentages were calculated based on pervious step; post-index period includes index date.

Of the 8,047 individuals with RTT, 43.6% (*n* = 3,505) were adults aged >20 years. When stratified by treatment status, approximately 25.5% (*n* = 392) of treated individuals and 47.8% (*n* = 3,113) of untreated individuals were >20 years of age (Figure 1). After applying the index dates and continuous enrollment, the final analytic sample included 1,820 RTT individuals (>20 years old): treated group (*n* = 290 [15.9%]) and untreated group (*n* = 1,530 [84.1%]).

### 3.2 Study measures

Baseline demographic and patient characteristics are reported in Table 1 for both groups. The mean (SD) age at RTT diagnosis was significantly lower in treated [28.8 (9.5) years vs. 31.8 (10.1) years] vs. untreated; similarly mean (SD) age at index treatment was significantly lower in treated [30.9 (9.9) years] vs. untreated [33.5 (10.0) years]. More than 90% were females in both groups, however, males were represented as well (5.5% vs. 6.7%). Differential diagnoses were observed to be significantly more frequent among treated vs. untreated: cerebral palsy (20.7% vs. 5.8%), non-specific developmental delay (11.0% vs. 1.6%), and autism spectrum disorder (9.7% vs. 2.9%), respectively. By geographic region, a significantly higher proportion of treated adults were from the South compared with untreated adults (35.9% vs. 29.3%, *p* < 0.05), while the proportion with unknown/unspecified region was significantly lower in the treated group (10.7% vs. 19.5%, *p* < 0.05).

**Table 1:** Demographics and characteristics among RTT adults treated versus untreated with trofinetide.

Demographics	Treated (*N* = 290)	Untreated (*N* = 1,530)	*p*-value
Age at RTT diagnosis (years)			
Mean, SD	28.8, 9.5	31.8, 10.1	
Median, IQR	26, 10	30, 15	<0.05
Age at TROF index (years)			
Mean, SD	30.9, 9.9	33.5, 10.0	
Median, IQR	28, 11	32, 15	<0.05
Age categories at TROF index, *n* (%)			
21–29	168 (57.9%)	640 (41.8%)	<0.05
30–39	76 (26.2%)	504 (32.9%)	<0.05
40–49	27 (9.3%)	258 (16.9%)	<0.05
≥50	19 (6.6%)	128 (8.4%)	0.36
Gender, *n* (%)			
Female	274 (94.5%)	1,427 (93.3%)	0.52
Male	16 (5.5%)	103 (6.7%)	0.52
Region, *n* (%)			
South	104 (35.9%)	448 (29.3%)	<0.05
Midwest	65 (22.4%)	299 (19.5%)	0.30
West	60 (20.7%)	335 (21.9%)	0.70
Northeast	30 (10.3%)	150 (9.8%)	0.86
Unknown/Unspecified	31 (10.7%)	298 (19.5%)	<0.05
Differential diagnosis*, *n* (%)			
Cerebral palsy	60 (20.7%)	88 (5.8%)	<0.05
Non-specific developmental delay	32 (11.0%)	25 (1.6%)	<0.05
Autism spectrum disorder	28 (9.7%)	45 (2.9%)	<0.05
Insurance type			
Third Party	142 (49.0%)	622 (40.7%)	<0.05
Medicare/Medicare Part D	72 (24.5%)	401 (25.4%)	0.79
Medicaid	61 (21.0%)	307 (20.1%)	0.77
Cash	8 (2.8%)	83 (5.4%)	0.08
Unknown	7 (2.4%)	117 (7.7%)	<0.05

IQR, Inter-quartile range; RTT, Rett syndrome; SD, Standard deviation.

^*^Other childhood disintegrative disorders and Angelman syndrome were <5%.

**Figure 2:**
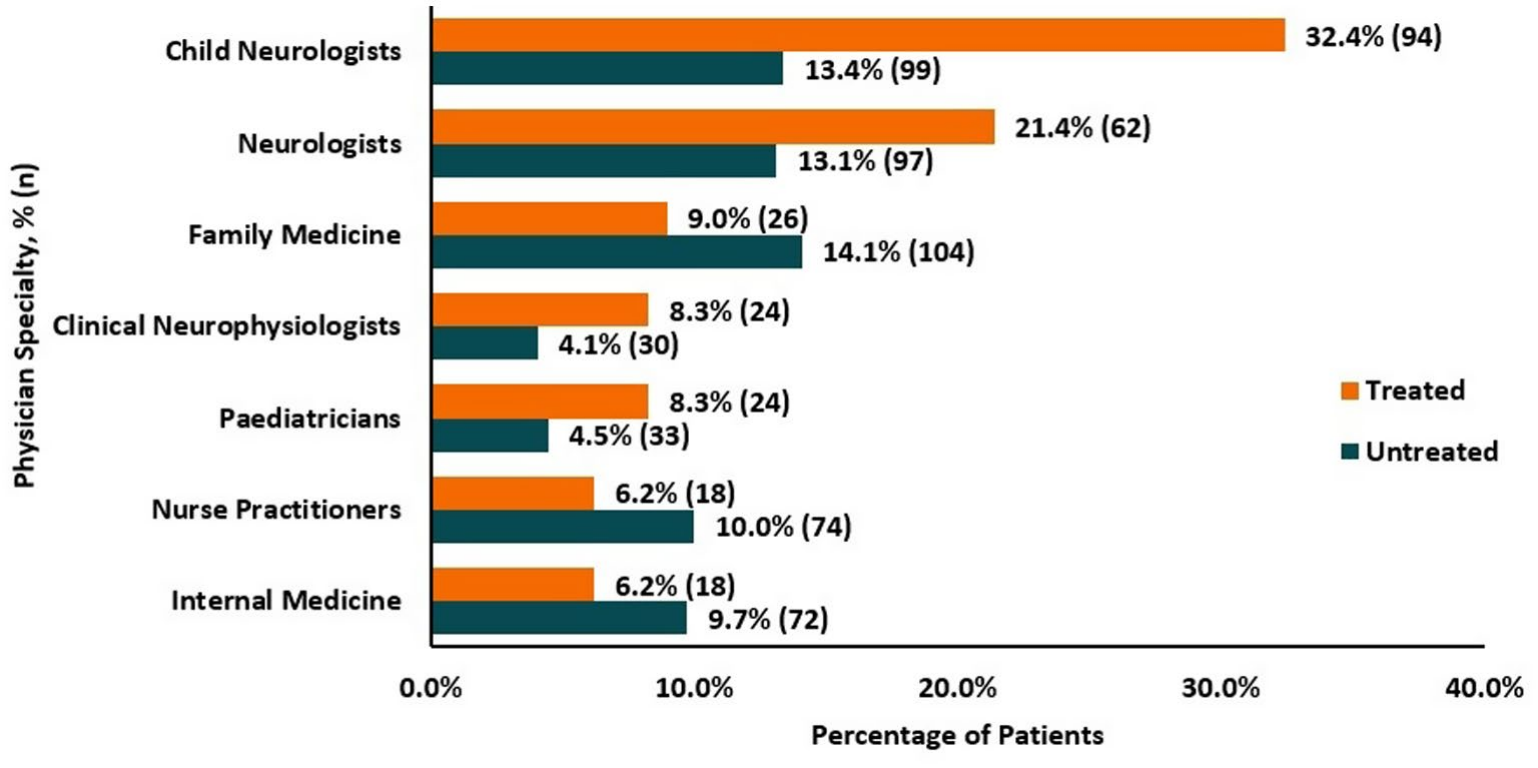
Physician specialty for adult individuals with RTT treated vs. untreated on trofinetide. Physician specialty was assessed closest to TROF index among treated and closest to RTT diagnosis among untreated. The percentages were calculated based on the available information regarding physician specialties among untreated (*n* = 739) and treated (*n* = 290) groups. The other known physician specialties include physician assistant, psychiatry, physical therapist, gastroenterologist etc.

Physician specialties were identified for all the treated group (*n* = 290); however, the untreated group has 51.7% (*n* = 791) unknown and 48.3% (*n* = 739) known specialties. Adults who were treated with TROF vs. untreated were managed frequently by child neurologists (32.4% vs. 13.4%), neurologists (21.4% vs. 13.1%), and clinical neurophysiologists (8.3% vs. 4.1%); however, treated to a lesser extent by family medicine practitioners (9.0% vs. 14.1%), nurse practitioners (6.2% vs. 10.0%) and internal medicine (4.1% vs. 9.7%) (Figure 2).

Table 2 provides baseline comorbidities and clinical characteristics during baseline for the treated adults vs. untreated adults. Overall, the comorbidities were very similar among the two groups, with small differences such as slightly higher rates of gastrostomy (25.9% vs. 21.0%), scoliosis (14.5% vs. 11.6%), and respiratory failure (10.7% vs. 9.9%) in the treated group; however, these differences were not statistically significant. The treated group had higher rates of RTT-related clinical features vs. the untreated group, loss of acquired communication skills (6.9% vs. 3.0%, *p* < 0.05) and prominent hand apraxia/dyspraxia (3.5% vs. 0.9%, *p* < 0.05) were shown to be statistically significant. Other RTT-related features, including breathing irregularities (5.9% vs. 4.9%) and nutritional deficiency (10.3% vs. 8.9%), were also numerically higher in the treated group; however, these differences were not statistically significant (Table 3).

**Table 2:** Comorbidities among RTT adults treated versus untreated with trofinetide.

Comorbidities	Treated (*N* = 290)	Untreated (*N* = 1,530)	*p*-value
Gastrointestinal disorders, *n* (%)			
Gastrostomy	75 (25.9%)	321 (21.0%)	0.08
Dysphagia	55 (19.0%)	283 (18.5%)	0.92
Constipation	35 (12.1%)	219 (14.3%)	0.36
Gastroesophageal reflux disorder	31 (10.7%)	163 (10.7%)	0.99
Vomiting	9 (3.1%)	47 (3.1%)	0.99
Infectious/viruses, *n* (%)			
Lower respiratory tract infection	18 (6.2%)	111 (7.3%)	0.61
Urinary tract infection	18 (6.2%)	78 (5.1%)	0.53
Upper respiratory infection	18 (6.2%)	46 (3.0%)	<0.05
Pyrexia	12 (4.1%)	44 (2.9%)	0.34
Musculoskeletal disorders, *n* (%)			
Scoliosis	42 (14.5%)	177 (11.6%)	0.19
Neurological disorders, *n* (%)			
Epilepsy	112 (38.6%)	567 (37.1%)	0.66
Seizures	43 (14.8%)	242 (15.8%)	0.74
Respiratory disorders, *n* (%)			
Respiratory Failure	31 (10.7%)	152 (9.9%)	0.78
Asthma	16 (5.5%)	76 (5.0%)	0.81
Cough	14 (4.8%)	83 (5.4%)	0.79
Aspiration	11 (3.8%)	79 (5.2%)	0.40

**Table 3:** RETT related features among RTT adults treated versus untreated with trofinetide.

Clinical features	Treated (*N* = 290)	Untreated (*N* = 1,530)	*p*-value
Growth/nutritional disorders, *n* (%)			
Nutritional deficiency	30 (10.3%)	136 (8.9%)	0.50
Growth abnormalities*	3 (1.0%)	28 (1.8%)	0.46
Neurodevelopmental disorders, *n* (%)			
Weakness/paralysis	33 (11.4%)	140 (9.2%)	0.28
Loss of acquired communication skills	20 (6.9%)	46 (3.0%)	<0.05
Behavioral disorders/symptoms**	18 (6.2%)	86 (5.6%)	0.80
Sleep dysfunction	11 (3.8%)	55 (3.6%)	0.99
Prominent hand apraxia/dyspraxia	10 (3.5%)	13 (0.9%)	<0.05
Wasting, dystonia, bradykinesia	4 (1.4%)	22 (1.4%)	0.99
Movement disorders	3 (1.0%)	9 (0.6%)	0.42
Stereotypic, repetitive hand movements	2 (0.7%)	5 (0.3%)	0.31
Loss of acquired motor skills	2 (0.7%)	13 (0.9%)	0.99
Respiratory disturbances, *n* (%)			
Breathing irregularities	17 (5.9%)	75 (4.9%)	0.59

* i.e., underweight/short stature; ** i.e., irritability, aggressive behavior, crying tantrums, self-mutilation, scratching, biting.

## 4 Discussion

This retrospective, real-world study is unique in helping understand the demographic and clinical characteristics of adults (>20 years) living with RTT in the United States and provides an insight into potential differences in the baseline characteristics among those treated with TROF vs. those untreated using linked specialty pharmacy and administrative claims data. In this analysis of real-world data, approximately one in two individuals with RTT had their observed RTT diagnosis after 20 years of age. These results, while consistent with prior US real-world evidence showing that 48% of individuals had an index RTT diagnosis at ≥18 years (7) suggest that the development of well-defined RTT diagnostic criteria as well as evolution in understanding the role of various MECP2 variants and increased genetic testing among children and alike may be a contributor to the diagnosis of RTT among adults.

The treated RTT adults were slightly younger at index than untreated adults; and both treated and untreated groups demonstrated a high multisystem comorbidity burden. Both treated and untreated RTT adults had similar baseline prevalence of concomitant comorbidities (e.g., dysphagia, epilepsy, gastrostomy, and scoliosis), reflecting the persistent neurologic and gastrointestinal burden that were observed among patients across RTT natural history studies and the TROF clinical program (4, 13, 22–24).

TROF treated adults, however, reported a higher frequency of documented prior differential diagnoses of diseases that may exhibit similar symptoms as RTT; specifically, 1 in 5 and 1 in 10 adults in the treated group, respectively, had at least one diagnostic claim for cerebral palsy and autism spectrum disorder compared to one in 20 and one in 30, respectively, among adults in the untreated group. This may reflect a more complex diagnostic journey reflecting the potential challenges in ruling out diseases that may resemble or mimic some of the core features of RTT. It is also possible that greater healthcare engagement and documentation among adults who initiate TROF, aligning with real-world “patient journey” literature describing prolonged diagnostic pathways and diagnostic overlap in RTT (7).

Provider specialty patterns further suggest that treated adults are frequently managed within pediatric-leaning or specialty neurology care, as reflected by higher proportions of index claims associated with child neurologists and neurologists. This finding is consistent with known challenges in the transition from pediatric to adult care for rare neurodevelopmental conditions, where specialized RTT expertise is often concentrated in pediatric centers (29). In this context, TROF initiation among adults may be occurring primarily where RTT expertise and familiarity with the clinical trial program are greatest.

Regional differences in TROF initiation were observed, including a higher proportion of treated adults in the South and a lower proportion with unknown or unspecified region. These findings may reflect differences in specialty-care access, provider familiarity, payer mix, referral patterns, caregiver awareness, or completeness of geographic information in the data. Because the study was descriptive, the underlying reasons for these regional differences could not be determined.

Despite TROF being indicated for individuals aged ≥2 years, our findings suggest that only approximately 1 in 5 eligible adults over 20 years of age received TROF treatment. This finding should be interpreted in the context of RTT as a lifelong neurodevelopmental disorder, with improved survival resulting in a growing population of adults living with RTT into middle age and beyond (9–13). Adult RTT care may differ from pediatric care because adults often have long-standing neurologic, gastrointestinal, respiratory, musculoskeletal, and functional complications, as well as caregiver needs and transition-of-care challenges that differ from pediatric care (13–17, 29). Therefore, understanding treatment patterns among adults is important to help inform adult-specific RTT care and future research. Several plausible reasons may explain the reasons for a sizable portion of adults remaining untreated with TROF. First, it is possible that most specialists who treat individuals with RTT may require a greater level of clinical experience in treating adults with RTT before they begin initiating TROF among the broader adult population with RTT. Second, it is possible that child neurologists and specialist pediatricians treating RTT may be more aware of TROF compared to adult neurologists or non-specialist neurologists or other providers. Third, it is also possible that adults with more clinically apparent or complex RTT presentations may be more likely to initiate TROF in early real-world practice. Finally, it is plausible that clinicians need published TROF data among adults to have greater confidence in prescribing it for adults.

Taken together, these findings underscore two parallel themes: (1) a substantial portion of adults with RTT remain untreated with TROF in real-world settings, suggesting considerable opportunity to understand and address barriers to initiation (e.g., transition-of-care gaps, coverage/access issues, provider familiarity, caregiver preferences), and (2) absence of data among adults over 20 years with RTT may create treatment hesitance among providers. While these findings suggest that both treated and untreated adults with TROF have a similar clinical comorbidity profile, it is possible that education about TROF are needed to assure adult neurologists who are yet to initiate TROF treatment. Furthermore, analysis examining TROF persistence in real-world settings among adults over 20 years are underway to provide additional insights about the potential real-world treatment effectiveness with TROF. Learnings from this study can provide more comfort with initiating TROF among adults with RTT.

The findings of this study should be interpreted in the context of limitations inherent to real-world claims analyses. Comorbidities and differential diagnoses were identified using ICD-10-CM diagnosis codes and may therefore be subject to miscoding or under-capture; however, RTT is a rare condition, and diagnostic coding may be relatively specific in specialty care settings. Physician specialty of each patient was assigned based on the claim closest to the index date and may not fully reflect longitudinal, multidisciplinary care involvement over time, particularly among adults whose care may be fragmented across providers and health systems. It is to be noted that the first documented RTT diagnosis, especially in claims data, may be influenced by factors such as delayed recognition, transition-of-care, changes in insurance coverage, or incomplete capture of earlier medical history. In addition, because this analysis focused on adults aged >20 years with available linked data, results may not be generalizable to pediatric RTT populations. The study population was identified using ICD-10-CM F84.2 diagnostic claims; genetic confirmation of MECP2 mutation status was not available in the data sources used. Therefore, the inclusion of clinically diagnosed cases without confirmatory genetic testing, or a small number of atypical RTT/RTT-like cases, cannot be ruled out. Despite these limitations, the use of a robust linked specialty pharmacy and claims dataset provides important real-world insight into an historically underserved adult RTT population. Future research should evaluate adult outcomes more comprehensively, including caregiver-reported measures, health care resource utilization, treatment persistence and dosing patterns. Given the limited availability of adult-specific TROF data, future investigations of adult RTT patients in real-world settings may be needed to examine if the magnitude of TROF effects differ among patients of varying phenotypes and severity.

## 5 Conclusion

In this real-world claims analyses of adults aged >20 years with Rett syndrome, only 16% of eligible adults initiated TROF while the majority remained untreated, highlighting a substantial opportunity to educate and use TROF in this underrepresented and clinically complex population. Overall, adults with RTT exhibited multisystem comorbidity burden among both groups, thus hesitancy in treating adult RTT should be alleviated. Treated adults also had higher documented rates of prior differential diagnoses and were more frequently managed by pediatricians or child neurology specialists, underscoring the potential influence of diagnostic complexity and transition-of-care dynamics on treatment initiation in adults. Taken together, these results suggest a vital need for: provider education about RTT among adult neurologists, transitions of care training among pediatric neurologists, and broader awareness about TROF as a treatment option for adults with RTT. Future research should evaluate adult-specific outcomes, longer-term persistence, dosing and titration strategies, and barriers to initiation to inform best practices and optimize long-term management across the broader adult RTT population.

## Data Availability

The raw data supporting the conclusions of this article will be made available by the authors, without undue reservation.
